# Strengths and limitations of new artificial intelligence tool for rare disease epidemiology

**DOI:** 10.1186/s12967-023-04152-0

**Published:** 2023-04-30

**Authors:** David Lapidus

**Affiliations:** LapidusData Inc., Oklahoma, OK USA

**Keywords:** Epidemiology, Incidence, Prevalence, Artificial intelligence, Rare diseases, Orphan drugs

## Abstract

The recent paper by Kariampuzha et al. describes an exciting application of artificial intelligence to rare disease epidemiology. The authors’ AI model appears to offer a major leap over Orphanet, the resource which is often a “first stop” for basic epidemiological data on rare diseases. To ensure appropriate use of this exciting tool, it is important to consider its strengths and weaknesses in context. The tool currently incorporates only PubMed abstracts, so key information located in the full text of articles is absent. Such missing information may include incidence and prevalence values, as well as important elements of study design and context. Additionally, results from the public version of the tool differ from those described in the original article, including obsolete values for prevalence and the use of non-prevalence studies in place of those listed in the article. At present, it would be appropriate to utilize the AI tool much like Orphanet: a helpful “first stop” which should be manually checked for completeness and accuracy. Users should understand the benefits of this exciting technology, and that it is not yet a panacea for the challenges of analyzing rare disease epidemiology.

**To the Editors:**

The recent paper by Kariampuzha et al. describes an exciting application of artificial intelligence to rare disease epidemiology. As an advisor to orphan drug companies on the commercial implications of rare disease epidemiology, I see first-hand the challenges that non-epidemiologists face when using epidemiology publications to make decisions about the development of rare disease therapies.

The authors’ AI model appears to offer a major leap over Orphanet, which is often a “first stop” for basic epidemiological data on rare diseases. In their case studies, the model quickly identified prevalence studies, and it summarized the results in more detail than the typical Orphanet tabular record.

This AI tool could increase the number of rare diseases for which concise summaries are available. This could improve resource allocation for drug development and other public health activities.

To ensure appropriate use of this exciting tool, its strengths and weaknesses must be understood. It is efficient: even an experienced epidemiologist would find it time-consuming to identify and summarize the data which this AI tool can output in mere minutes.

Several weaknesses should also be considered from the perspective of its likely users. (This tool is clearly not intended to replace an experienced epidemiologist; it should not be judged against an unrealistic standard.)

The tool analyzes a limited data source which may bias its results. Its “universe” consists only of PubMed abstracts. These contain a fraction of all published epidemiology data. The tool does not analyze articles’ full text, which may have essential context that could affect the interpretation of data in the abstract. Some full text articles are freely available; a future iteration of the tool might incorporate these sources in place of their abstracts.

For paid articles, it is unclear how a future version of the tool might access their full text without a vast number of licensing agreements. This is unfortunate, given that much epidemiology information resides in paid articles: a cursory examination of articles on the epidemiology of phenylketonuria reveals that only one-quarter are classified as “free” in PubMed.

Another concern about missing information arises because the tool is limited to PubMed. Future iterations of the tool could be improved by incorporating other literature databases.

At a higher level, these issues can be described as a lack of “sensitivity.” Users of the tool must understand that its outputs may miss key data. While an epidemiologist may consider this an obvious limitation, it is important to remember that many general users of this seemingly authoritative tool (as with Orphanet) are not aware that it may be missing data that could impact their decision-making.

In addition to the potential bias from limited data sources, the tool also appears to perform inconsistently even within its defined “universe.” In the paper, Kariamphuza et al. showed the tool’s output for fibrodysplasia ossificans progressiva (FOP): a paper by Baujat et al. It correctly summarized the topline results; this paper is the gold standard for FOP prevalence, so this is encouraging.

However, a query of the public version of the tool for the same disease using broad criteria (up to 1000 results with “lenient” inclusion criteria) returned three articles, but not Baujat 2017. The three results are case studies, not prevalence studies. Their abstracts include a prevalence statistic cited from older sources, but the value they reported is obsolete to Baujat’s study. A user of the tool would gain an incorrect impression of the prevalence of FOP.

Like Baujat 2017, other FOP prevalence studies were absent, such as Pignolo 2021, Morales-Piga 2012, and Connor 1982. Thus, for FOP, the public version of the tool extracted obsolete results from case study articles, and it did not capture any of the actual prevalence studies.

Another disease for which the public version of the tool did not perform as expected is autoimmune pulmonary alveolar proteinosis (aPAP). Several aPAP prevalence studies have been published, but none were captured by the tool when using inclusive criteria. The tool’s sole output was a review article, and it extracted an epidemiology statistic which is consistent with the actual studies of aPAP prevalence. Still, the absence of any prevalence studies from the output is surprising.

The cases of FOP and aPAP suggest some caution when interpreting the results of this AI tool. Of course, the rapid improvement of AI models is to be expected, and we may hope that Kariamphuza et al. will soon offer the planned iterations described in their paper, and perhaps other improvements, too. For now, it would be appropriate to utilize the AI tool much like Orphanet: a helpful “first stop” which should be manually checked for completeness and accuracy. Users should understand the benefits and limits of this exciting technology, and that it is not yet a panacea for the challenges of analyzing rare disease epidemiology.

## Data Availability

Can be provided upon request.
